# Phase I clinical trial of the combination of eribulin and everolimus in patients with metastatic triple-negative breast cancer

**DOI:** 10.1186/s13058-019-1202-4

**Published:** 2019-11-08

**Authors:** Jin Sun Lee, Susan E. Yost, Suzette Blanchard, Daniel Schmolze, Hongwei Holly Yin, Raju Pillai, Kim Robinson, Aileen Tang, Norma Martinez, Jana Portnow, Wei Wen, John H. Yim, Heather Ann Brauer, Yuqi Ren, Thehang Luu, Joanne Mortimer, Yuan Yuan

**Affiliations:** 1 Department of Medical Oncology & Therapeutics Research, City of Hope National Medical Center and Beckman Research Institute, 1500 E. Duarte Road, Duarte, CA 91010 USA; 2 Department of Biostatistics, City of Hope National Medical Center and Beckman Research Institute, Duarte, CA USA; 3 Department of Pathology, City of Hope National Medical Center and Beckman Research Institute, Duarte, CA USA; 4 Department of Surgery, City of Hope National Medical Center and Beckman Research Institute, Duarte, CA USA; 5 NanoString Technologies, Inc., Seattle, WA USA; 6 OncoGambit, Irvine, CA USA

**Keywords:** Phase I trial, Eribulin, Everolimus, Metastatic TNBC

## Abstract

**Background:**

Alteration of the PI3K/AKT/mTOR pathway is a common genomic abnormality detected in triple-negative breast cancer (TNBC). Everolimus acts synergistically with eribulin in TNBC cell lines and xenograft models. This phase I trial was designed to test the safety and tolerability of combining eribulin and everolimus in patients with metastatic TNBC.

**Methods:**

The primary objective of this study was to evaluate the safety and toxicities of the combination. Patients with metastatic TNBC who had up to four lines of prior chemotherapies were enrolled. The combination of eribulin and everolimus was tested using three dosing levels: A1 (everolimus 5 mg daily; eribulin 1.4 mg/m^2^ days 1 and 8 every 3 weeks), A2 (everolimus 7.5 mg daily; eribulin 1.4 mg/m^2^, days 1 and 8 every 3 weeks), and B1 (everolimus 5 mg daily; eribulin 1.1 mg/m^2^ days 1 and 8 every 3 weeks).

**Results:**

Twenty-seven patients with median age 55 years were enrolled. Among 8 evaluable patients who received dose level A1, 4 had dose-limiting toxicities (DLTs). Among 3 evaluable patients treated with dose level A2, 2 had DLTs. Among 12 evaluable patients who received dose level B1, 4 had DLTs. The DLTs were neutropenia, stomatitis, and hyperglycemia. Over the study period, 59% had a ≥ grade 3 toxicity, 44% had ≥ grade 3 hematologic toxicities, and 22% had grade 4 hematologic toxicities. The most common hematological toxicities were neutropenia, leukopenia, and lymphopenia. Thirty-three percent had grade 3 non-hematologic toxicities. The most common non-hematological toxicities were stomatitis, hyperglycemia, and fatigue. The median number of cycles completed was 4 (range 0–8). Among 25 eligible patients, 9 patients (36%) achieved the best response as partial response, 9 (36%) had stable disease, and 7 (28%) had progression. The median time to progression was 2.6 months (95% CI [2.1, 4.0]), and median overall survival (OS) was 8.3 months (95% CI [5.5, undefined]).

**Conclusion:**

Eribulin 1.1 mg/m^2^ days 1 and 8 every 3 weeks with everolimus 5 mg daily was defined as the highest dose with acceptable toxicity (RP2D). The combination is safe, and efficacy is modest. A post hoc analysis showed that participants that used dexamethasone mouthwash stayed on treatment for one additional cycle.

**Trial registration:**

ClinicalTrials.gov, NCT02120469. Registered 18 April 2014

## Background

Triple-negative breast cancer (TNBC) is an aggressive subtype of breast cancer characterized by a lack of estrogen receptor, progesterone receptor, and human epidermal growth factor receptor 2 (HER-2) amplification and accounts for approximately 15 to 20% of all breast cancer [1–3]. TNBC is a heterogeneous disease comprised of several molecular subtypes defined by gene expression profiling [4–7]. TNBCs often become chemotherapy-resistant upon relapse; hence, clinical outcome for patients with metastatic TNBC (mTNBC) is particularly poor with a median overall survival (OS) of approximately 13–16 months, largely due to the lack of effective targeted therapy [2, 8, 9]. There have been recent breakthrough therapies in subpopulations of patients with mTNBC, such as poly ADP ribose polymerase (PARP) inhibitors olaparib and talazoparib in patients with germline BRCA1 or BRCA2 mutation [10–12]. Immune checkpoint inhibitors targeting programmed death 1 (PD-1) or programmed death ligand 1 (PD-L1) showed efficacy in PD-L1-positive TNBCs [13–18]. Despite the progress being made, there is an unmet need for novel targeted therapies to improve the outcome of patients with mTNBC.

Phosphatidylinositol 3-kinase (PI3K)/AKT pathway alterations are some of the most common genomic alterations identified in breast cancer, and recently, the FDA granted approval of PI3Kα-specific inhibitor alpelisib plus fulvestrant in PIK3CA-altered ER-positive metastatic breast cancer [19]. The PI3K/AKT pathway alterations include the loss-of-function of the tumor suppressor phosphatase and tensin homolog (PTEN) [20, 21]. Overall, the activating mutations of PI3K catalytic subunit alpha (*PIK3CA*)/*AKT*/*PTEN*-altered tumors account for approximately 35% of patients with TNBC [21, 22]. It is also noted that the alteration of this pathway increases in metastatic disease, which suggests that PI3K pathway alterations may be associated with chemotherapy resistance in TNBC [23, 24].

Eribulin mesylate is a synthetic analog of halichondrin B, a natural product isolated from the marine sponge *okadai kadota* [25]*.* It shows antitumor activity via a tubulin-based anti-mitotic mechanism, which leads to G2/M cell cycle arrest, disruption of mitotic spindles, and apoptotic cell death. Eribulin inhibits cell growth in a wide range of cancer cell lines including breast, colon, prostate, and ovarian cancer [26]. Eribulin suppresses metastasis of breast cancer cells by reversing the phenotype from epithelial-mesenchymal transition (EMT) to mesenchymal-epithelial transition (MET) states [27]. In the EMBRACE trial, eribulin showed OS benefit compared with conventional single-agent chemotherapy of physician’s choice in heavily pretreated patients with metastatic breast cancer [28]. This result led to FDA approval in 2011.

Everolimus inhibits cytokine and growth factor-dependent cell proliferation by inhibiting the mammalian target of rapamycin (mTOR), a key protein involved in the PI3K pathway [29]. In both preclinical models and early clinical trials in breast cancer, everolimus demonstrated promising efficacy [30, 31]. Furthermore, a randomized phase III study has indicated better clinical outcomes for the treatment of metastatic hormone receptor-positive breast cancer in combination with an aromatase inhibitor [32]. Paradoxically, single-agent mTOR inhibition with everolimus enhances upstream receptor tyrosine kinase activity and activates downstream AKT activity [33, 34], which may explain the lack of activity as a single agent in breast cancer. Increased efficacy has been observed when everolimus is combined with HER2-targeted therapy [35–37] or carboplatin [38, 39].

Our preclinical studies demonstrated the synergistic effect of eribulin and everolimus in TNBC cell lines and murine models [34]. In TNBC cell lines MDA-MB-468 and BT549, phosphorylation of AKT was suppressed by eribulin. The combination of eribulin and everolimus resulted not only in an increased reduction of p-S6K1 and p-S6, but also a synergistic inhibition of cell survival in vitro and enhanced suppression of tumor growth in two mouse models. These findings provide a preclinical foundation for targeting both the microtubule cytoskeleton and the PI3K/AKT/mTOR pathway in the treatment of refractory TNBC [34].

We hypothesize that the combination of eribulin and everolimus may be effective in patients with metastatic TNBC resistant to anthracyclines and taxanes. Here, we report the results of a phase I trial of everolimus and eribulin in patients with mTNBC.

## Methods

### Study design

This study was a single-center phase I trial conducted in patients with mTNBC resistant to anthracycline and/or taxane. The primary objective was to evaluate the safety and tolerability of everolimus and eribulin and to determine the recommended phase 2 dose (RP2D) of the combination in patients with mTNBC who had received up to four lines of prior chemotherapy for metastatic disease. The secondary objective was to assess the activity based on the response rate (RR) and progression-free survival (PFS). Study treatment was provided until disease progression or unacceptable toxicity. The study was conducted with the Declaration of Helsinki and Good Clinical Practice. This study is registered at the clinical trial website ClinicalTrials.gov under number NCT02723877.

### Patient population

This study was conducted between November 2014 and March 2019 with an institutional review board (IRB) approval at the City of Hope National Cancer Center. Informed consent forms were signed by all patients prior to study entry. Main patient eligibility criteria included the following: age ≥ 18 years; life expectancy of ≥ 3 months; ECOG performance status 0–2; histologically confirmed TNBC (ER < 10%, PR < 10%, HER-2neu-negative defined by IHC 0 or 1, or FISH-negative); 0–3 lines of prior chemotherapy for metastases; prior treatment with anthracycline and/or taxane therapy including (neo) adjuvant setting; adequate bone marrow reserve (hemoglobin ≥ 9 g/dL, absolute neutrophil count (ANC) ≥ 1500/mm^3^, platelet count ≥ 100,000/mm^3^); adequate renal function defined by creatinine ≤ 1.5× the upper limit of normal (ULN); adequate liver function defined by total bilirubin ≤ 1.5× ULN, alanine transaminase (ALT), and aspartate transaminase (AST) ≤ 2.5× ULN (≤ 5× ULN in patients with liver metastases); able to obtain baseline computed tomography (CT), bone scan, or positron emission tomography (PET)/CT; and able to provide written informed consent. The mandatory use of dexamethasone mouthwash (alcohol-free 0.5 mg/5 mL dexamethasone solution) was amended at time of dose level B1 to reduce oral mucositis based on SWISH study results [40].

The main exclusion criteria included the following: chemotherapy or radiotherapy within 2 weeks prior to study entry; persisting ≥ grade 2 AE defined by NCI CTCAE v4.0 at screening visit except alopecia; untreated or unstable brain metastases; prior eribulin or everolimus use; HIV; chronic hepatitis B or C (known from the existing medical record); concomitant use with strong or moderate CPY3A4/PgP inhibitors and CPY3A4/PgP inducers; uncontrolled current illness including, but not limited to, ongoing or active infection (≥ grade 2 based on the NCI CTCAE v4.0); symptomatic congestive heart failure, unstable angina pectoris, or myocardial infarction within the past 6 months; cardiac ventricular arrhythmias requiring anti-arrhythmic therapy; unable to take oral medication; history of non-compliance to medical regimens; and psychiatric illness or social situations that would limit compliance with study requirements.

### Baseline assessments

At screening visit, physical examination, vital signs, and labs including complete blood cell count (CBC) and differential, complete metabolic panels (CMP), and liver function test (LFT) were performed and repeated every 3 weeks on day 1 of each cycle. A baseline electrocardiogram (EKG) was also obtained and repeated as clinically indicated. All patients were screened for hepatitis B and C infection. Urine or serum pregnancy test was also performed at screening. For tumor response assessment, CT scan of the chest/abdomen/pelvis and bone scan were performed for response evaluation criteria in solid tumor (RECIST1.1) evaluation. Brain MRI with contrast was used if clinically indicated.

### Study treatment

Three dosing levels of the combinations were tested. For dose level A1, everolimus 5 mg daily and eribulin 1.4 mg/m^2^ days 1 and 8 every 3 weeks were given. For dose level A2, everolimus 7.5 mg daily and eribulin 1.4 mg/m^2^ days 1 and 8 every 3 weeks were given. In dose level B1, everolimus 5 mg daily and eribulin 1.1 mg/m^2^ days 1 and 8 every 3 weeks were given. A mandatory dexamethasone mouthwash consisting of 10 mL of alcohol-free dexamethasone 0.5 mg/5 mL oral solution (swish for 2 min and spit, four times daily) was added to the protocol in an amendment in 2017 when dose level B1 was opened [40]. Other supportive medications, including anti-emetics, were used according to the current standard of care guidelines. Patients were treated until progression or unacceptable toxicity occurred.

### Statistical methods

The study utilized the toxicity equivalence range (TEQR) design with a target equivalence range for dose-limiting toxicities (DLTs) of 0.20–0.35. Toxicity levels of ≥ 0.51 were considered too toxic, and the dose that achieve this level was closed. Patients entered the protocol in cohorts of 3. This trial was considered complete when 12 evaluable patients were studied at a single dose level with a toxicity level of < 0.51. The RP2D was determined as the dose closest to the target of 0.25 below 0.51 based on isotonic regression [39].

DLT was defined as (i) grade 3 febrile neutropenia, grade 3 neutropenia lasting for > 7 days, grade 4 neutropenia, and grade 4 thrombocytopenia; (ii) any non-hematological toxicity ≥ grade 3, controllable grade 3 nausea and vomiting, < 5 days of grade 3 fatigue, triglycerides < 1500 mg/dL which recovers in 1 week, grade 3 lab abnormalities that are correctable to grade 2 or less with 24 h, and grade 3 hyperglycemia that is controlled to grade 2; (iii) treatment delays > 2 weeks as a result of unresolved toxicity during the first cycle of therapy; and (iv) failure to complete at least 75% of planned dose of either drugs during cycle 1 due to toxicity. Toxicity was evaluated based on the National Cancer Institute Common Terminology Criteria for Adverse Events (NCI CTCAE) v4.0.

Descriptive statistics were used to summarize the patient demographic characteristics and adverse events. Rates and exact Clopper-Pearson 95% confidence intervals were provided for DLTs at the RP2D and disease response. PFS and OS were described using Kaplan-Meier methods.

### Tumor genomic profiling

Formalin-fixed paraffin-embedded (FFPE) tumor tissues from patients who consented to tumor tissue analysis were collected. DNA exome sequencing was performed using the commercially available assay FoundationOne® [41] (*n* = 9). For tumor RNA profiling, NanoString nCounter® PanCancer Pathway (*n* = 20) and Breast Cancer 360™ Panel (BC360™) (*n* = 11) were performed for patients who had sufficient baseline FFPE tumor tissue. PanCancer multiplex gene expression analysis was performed using 770 genes from cancer-associated canonical pathways including: MAPK, STAT, PI3K, RAS, cell cycle, apoptosis, Hedgehog, Wnt, DNA damage control, transcriptional regulation, chromatin modification, and TGF-β. BC360™ panel analysis was performed for 770 genes across 24 key breast cancer pathways and processes (PAM50 signature, TNBC signature, claudin-low signature, tumor inflammation signature, and 38 unique signatures with well-established roles in breast cancer and immuno-oncology) [42]. Tumor sections were microdissected from the patients’ unstained slides, and RNA was extracted using miRNeasy FFPE kit (Qiagen). RNA concentration was assessed with the Nanodrop spectrophotometer ND-1000 and Qubit 3.0 fluorometer (Thermo Scientific, CA). RNA fragmentation and quality control were further determined by 2100 Bioanalyzer (Agilent, CA). RNA was hybridized with codeset from gene panel at 65 °C for 16–20 h. Post-hybridization probe-target mixture was purified and quantified with nCounter Digital Analyzer, and all data analysis was performed on nSolver (NanoString Technologies, WA). All raw data from expression analysis were first aligned with internal positive and negative controls then normalized to the selected housekeeping genes included in the assay. Differential gene expression patterns as well as pathway and cell type scores with statistical analyses were performed with nSolver software (NanoString Technologies, WA). BC360™ signatures including PAM50 breast cancer intrinsic subtype classifier and the tumor inflammation signature were analyzed by NanoString. NanoString data was analyzed for statistical significance using nSolver analysis software. Box plots of differential expression with associated *p* value calculation were generated using Graphpad Prism 5.0 software (La Jolla, CA, USA).

## Results

**Fig. 1:**
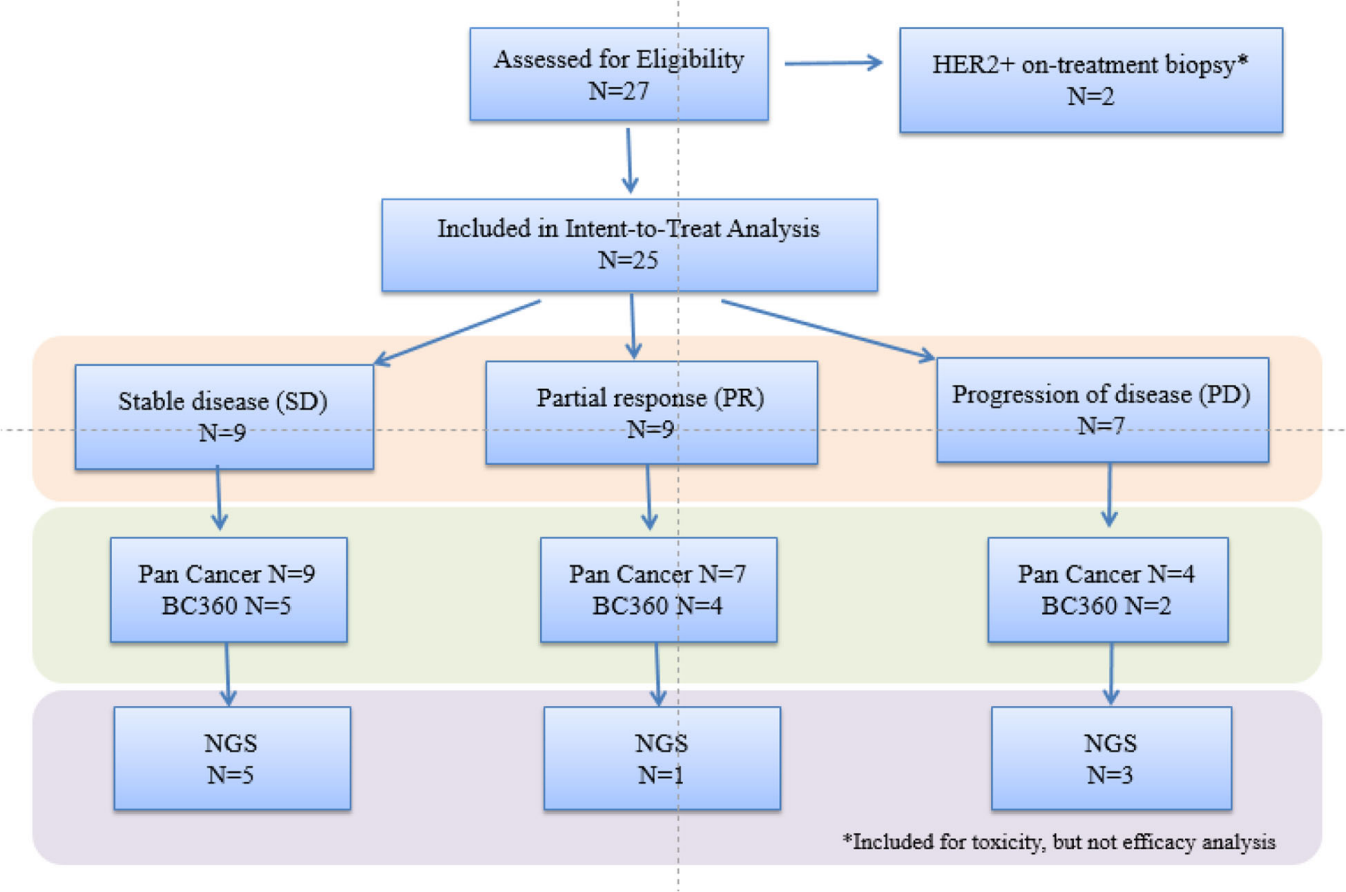
Patient accrual and correlative analysis summary. A total of 27 patients were accrued and received treatment on the study. Two of the patients’ on-treatment biopsy revealed HER2+ FISH-amplified tumor, and study treatment was terminated (patients were excluded from the efficacy analysis but included in the toxicity analysis). mRNA profiling was performed for 20 patients with NanoString PanCancer Pathways analysis and 11 patients for BC360™. FoundationOne ® genomic mutation profiles were available for 9 patients

A total of 27 patients were enrolled and received treatment from November 2014 to March 2019. The median age of patients was 55 (range 36–76). Sixty-seven percent (18/27) of participants were non-Hispanic and 33% (9/27) were Hispanic (Table 1). Sites of metastases were distant lymph nodes 67% (18/27), lung 56% (15/27), distant skin/subcutaneous tissue 41% (11/27), bone 41% (11/27), liver 37% (10/27), pleura 33% (9/27), brain 7% (2/27), and others 19% (5/27). The number of patients receiving 0–1 lines of chemotherapy for metastases was 44% (12/27), and the number of patients receiving ≥ 2 lines of chemotherapy was 56% (15/27). Two of 27 patients were found to be HER-2/neu-amplified at the time of confirmatory biopsy and were taken off study. These two patients were not included in efficacy analysis but were included in toxicity analysis (Fig. 1).

**Table 1:** Patient characteristics

	Patients (*N* = 27)
Age	55 (36–76)
ECOG performance status	
0	8 (30%)
1	15 (56%)
2	4 (15%)
Median BMI (range)	30 (19–48)
Race/ethnicity	
White	18 (67%)
Asian/Pacific Islander	3 (11%)
Black	2 (7%)
Unknown	4 (15%)
Non-Hispanic	18 (67%)
Hispanic	9 (33%)
Initial tumor stage	
I	3 (11%)
II	14 (52%)
III	6 (22%)
IV	4 (15%)
Lines of chemo for metastases	
0–1	12 (44%)
≥ 2	15 (56%)
Sites of metastases	
Distant lymph nodes	18 (67%)
Lung	15 (56%)
Distant skin/subcutaneous tissue	11 (41%)
Bone	11 (41%)
Liver	10 (37%)
Pleura	9 (33%)
Brain	2 (7%)
Others*	5 (19%)

*Others are the latissimus dorsi muscle, adrenal glands, ovary, and contralateral breast

### Treatment

Three dose levels of everolimus and eribulin were evaluated: level A1 (everolimus 5 mg daily; eribulin 1.4 mg/m^2^ on days 1 and 8 every 3 weeks), level A2 (everolimus 7.5 mg daily; eribulin 1.4 mg/m^2^ on days 1 and 8 every 3 weeks), and level B1 (everolimus 5 mg daily; eribulin 1.1 mg/m^2^ on days 1 and 8 every 3 weeks) (Table 2). Initially, 4 patients received treatment on dose level A1. One patient was not evaluable for dose escalation, due to receiving < 75% drug as a result of an event that was unrelated to treatment. There were no DLTs in 3 evaluable patients, so the dose was escalated based on the guidelines for the TEQR design. Four patients were treated on dose level A2. One patient was found to be HER2+ and was taken off study. A total of 2/3 evaluable patients experienced DLTs in cycle 1, so dose level A2 was de-escalated to dose level A1 according to TEQR guidelines. Six additional patients were treated at dose level A1. Of those, 1 patient progressed without completing the first cycle and did not have a DLT, hence unevaluable for dose escalation. Of the 5 evaluable patients, 4 experienced DLTs. Of the 8 patients evaluable at dose level 1, 4/8 had DLTs (50%). The DLTs included 2 patients with grade 4 neutropenia, 1 patient with grade 3 mucositis, and 1 patient received less than 75% of planned dose of everolimus due to persisting grade 2 mucositis. The protocol was amended to add a lower dose level of B1, eribulin at 1.1 mg/m^2^ on days 1 and 8 every 3 weeks and everolimus 5 mg daily. Thirteen patients were treated at dose level B1. One patient was ineligible due to HER2+ disease on repeat of biopsy. Of the 12 patients treated, the DLT rate at dose level B1 was 33% (4/12) with a 95% CI of 0.1, 0.65, thus completing the phase 1 part of the trial (Table 2). Dose level B1 (everolimus 5 mg daily and eribulin 1.1 mg/m^2^ on days 1 and 8 every 3 weeks) was determined to be the RP2D doses. The median number of cycles completed was 4 (0–18).

**Table 2:** Dosing levels and DLTs

Dosing levels	Number	Evaluable	DLT (%)	DLT type
A1: everolimus 5 mg daily; eribulin 1.4 mg/m^2^ days 1 and 8 every 3 weeks	10	8^†^	4 (50%)	1 patient: grade3 mucositis 2 patients: grade 4 neutropenia, including one with febrile neutropenia 1 patient: received less than 75% of planned dose of everolimus due to prolonged grade 2 mucositis
A2: everolimus 7.5 mg daily; eribulin 1.4 mg/m^2^ days 1 and 8 every 3 weeks	4	3*	2 (67%)	1 patient: grade 3 hyperglycemia 1 patient: grade 3 mucositis
B1: everolimus 5 mg daily; eribulin 1.1 mg/m^2^ days 1 and 8 every 3 weeks	13	12‡	4 (33%)	2 patients: grade 4 neutropenia 1 patient: grade 3 mucositis 1 patient: did not receive 75% of everolimus due to persisting grade 2 mucositis

^†^One patient progressed without completion of the first 2 cycles of therapy, hence unevaluable for DLT; 1 patient did not receive planned dose due to grade 3 hypoglycemia attributed to diabetes

*One patient was found to be HER2+ on repeat biopsy, deem ineligible

^‡^One patient was found to be HER2+ on repeat biopsy, deem ineligible

### Dose modification

Sixty-eight percent (17/25) of participants had a dose modification or hold, including 56% (14/25) for eribulin and 60% (15/25) for everolimus.

### Toxicities

Of the 27 patients, 96% (26/27) had a grade ≥ 2 toxicity, and 59% (16/27) had grade 3–4 toxicity attributed to treatment. There were no grade 5 toxicities attributed to treatment.

#### Hematologic toxicities

**Fig. 2:**
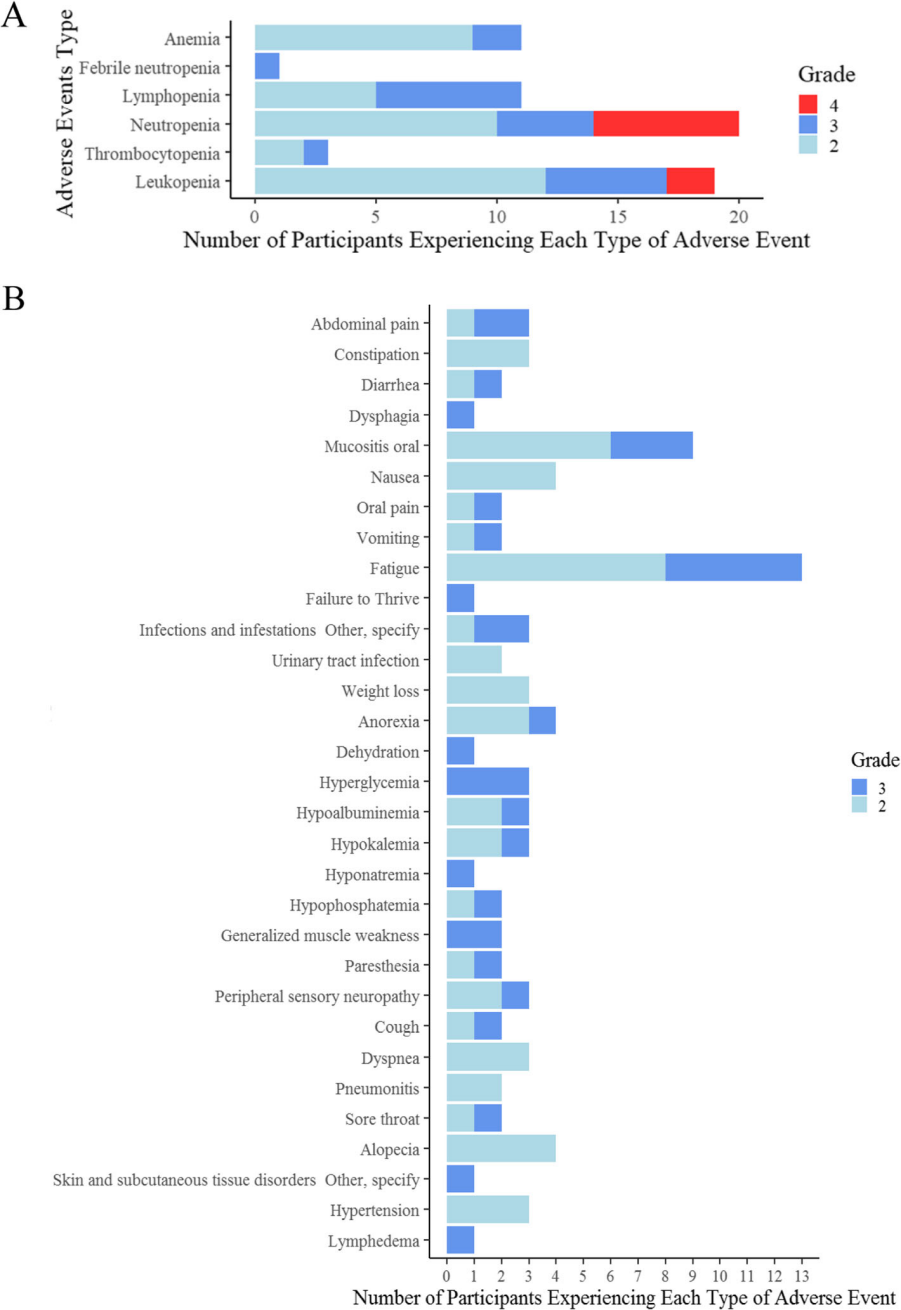
Summary of toxicities. **a** Hematological toxicities: 12/27 (44%) had ≥ grade 3 hematological toxicity, including neutropenia (*n* = 10), lymphopenia (*n* = 6), and leukopenia (*n* = 7). **b** Non-hematologic toxicities: 9/27 (33%) had grade 3 non-hematological toxicity, including oral mucositis (*n* = 3), hyperglycemia (*n* = 3), and fatigue (*n* = 5). The counts of maximum ≥ grade 2 for each participant was listed for each event type (either ≥ grade 3 toxicity, or 2 participants experienced a grade 2 toxicity)

Of the 27 patients, 44% (12/27) had grade 3 and above hematologic toxicities, including neutropenia (*n* = 10), lymphopenia (*n* = 6), and leukopenia (*n* = 7) (Fig. 2a).

#### Non-hematologic toxicities

Of the 27 patients, 9/27 (33%) had grade 3 non-hematologic toxicities, including oral mucositis (*n* = 3), hyperglycemia (*n* = 3), and fatigue (*n* = 5). The counts of maximum grade 2 and above for each participant were listed for each event type where either a grade 3 or above toxicity was experienced, or 2 participants experienced a grade 2 (Fig. 2b). When dose level B1 was opened, a mandatory dexamethasone (Dex) mouthwash was added to reduce oral mucositis based on principle of best medical practice and clinical trial data [40]. Of the 12 evaluable participants enrolled to dose level B1, 2 did not use Dex mouthwash and 3 of the patients in the A1 dose used Dex mouthwash. A post hoc analysis was performed to compare the number of cycles that patients received in the Dex group with the non-Dex group. The median number of cycles was higher by 1 cycle (from 3 to 4 cycles) in the participants that took Dex. This was statistically significant using a Wilcoxon rank sum test (Dex: *n* = 13; non-Dex: *n* = 12; *p* = 0.046).

### Antitumor activity and survival

**Fig. 3:**
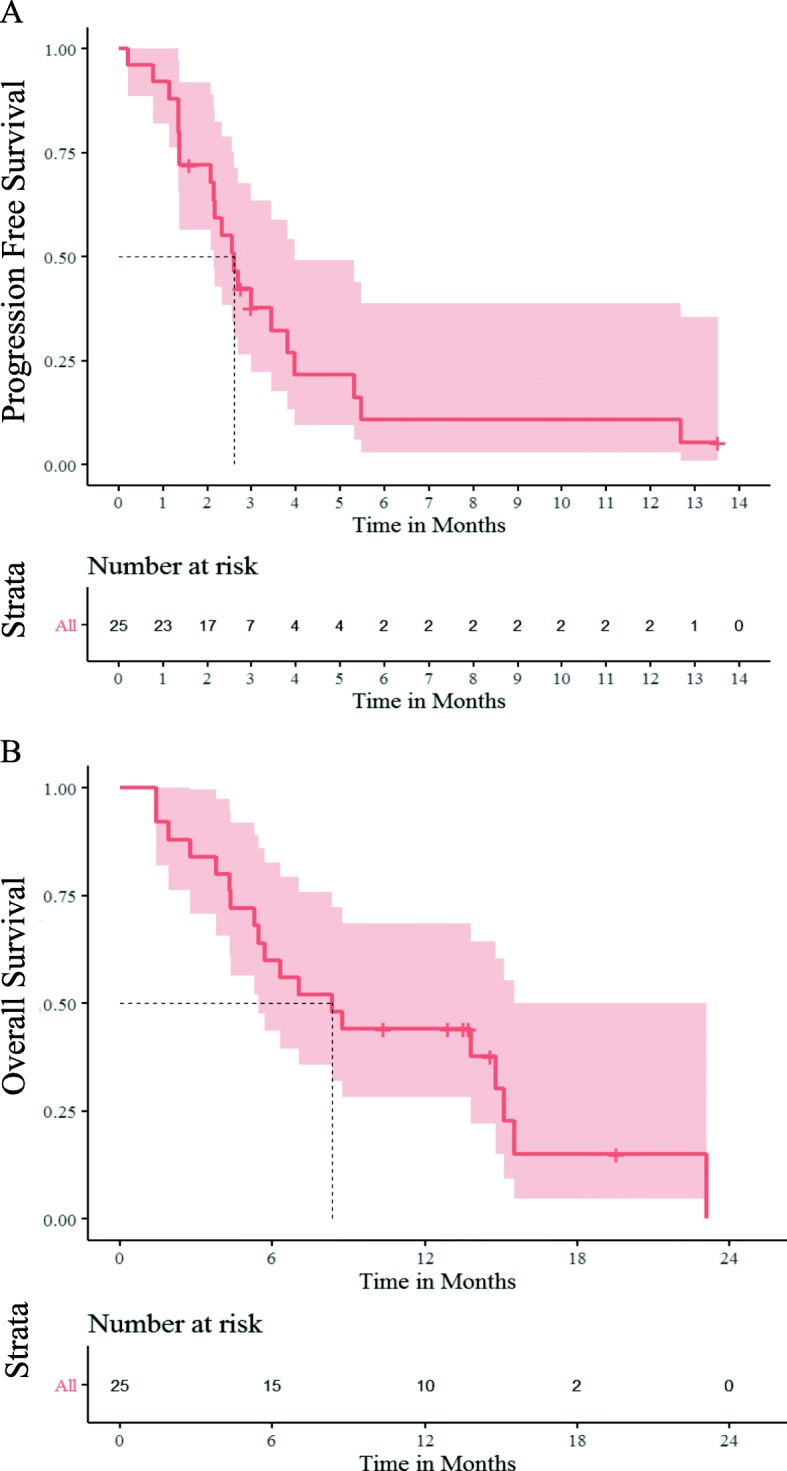
Kaplan-Meier survival analysis. **a** Median PFS was 2.6 months (95% CI [2.1, 4.0]). **b** Median OS was 8.3 months (95% CI [5.5, undefined])

Of the 25 evaluable patients, 9/25 (36%) had a partial response (PR), 9/25 (36%) achieved a best response of stable disease (SD), and 7/25 (28%) had progression of disease (PD). A total of 21/25 (84%, 95%CI [64%, 95%]) experienced progression by RECIST1.1 or showed clinical progression. The median PFS was 2.6 months (95%CI [2.1, 4.0]). Nineteen of 25 (76%, 95% CI [55%, 91%]) eligible participants had event of death at the time of data cutoff (25 March 2019), and the median OS was 8.3 months (95% CI [5.5, undefined]). The cause of death was disease progression for 18 and failure to thrive for 1. Kaplan-Meier curves for PFS and OS are shown in Fig. 3. Of the 24 participants that were off treatment, 17 (71%) were off for progression, 4 (17%) for clinical progression, 2 (8%) for toxicity, and 1 (4%) for lack of insurance coverage.

### mRNA expression and response

**Fig. 4:**
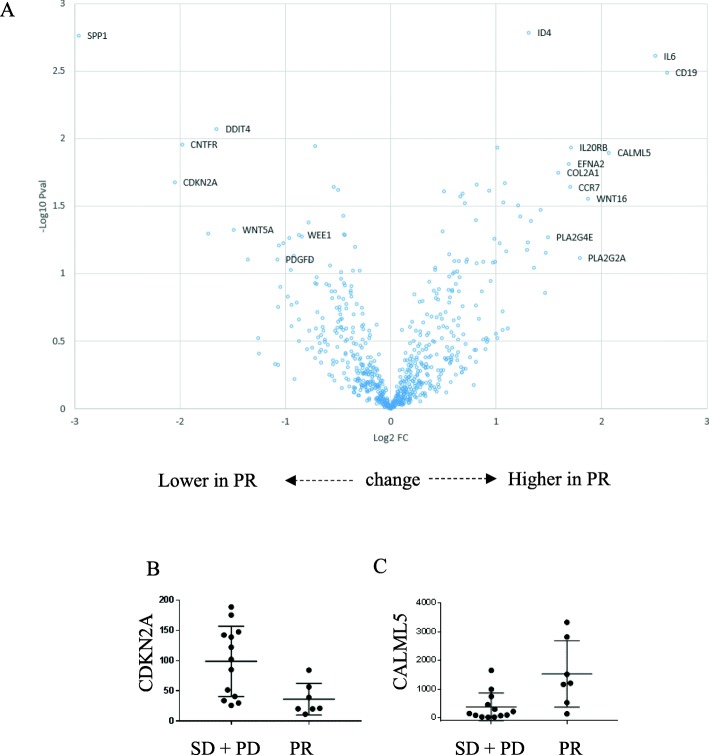
NanoString PanCancer Pathways® analysis (*n* = 20). **a** Volcano plot showing differentially expressed genes with linear fold change > 2 and *p* < 0.05 comparing SD+PD and PR. **b** Decreased CDKN2A expression (*p* = 0.02) in responders. **c** Increased CALML5 expression (*p* = 0.01) in responders

NanoString PanCancer Pathway profiling was performed for 20 patients who had sufficient FFPE from metastatic biopsy. PanCancer Pathway with the grouping of patients’ best response analysis revealed a total of 22 differentially expressed genes (DEGs) comparing PR (*n* = 7) vs. SD+PD (*n* = 13) (linear fold change > 2 and *p* < 0.05). Five DEGs (CDKN2A, WNT5A, CNTFR, DDIT4, and SPP1) were downregulated in PR, and 17 DEGs were upregulated in PR. Of the 22 genes, 9 DEGs are involved in the PI3K pathway (Table 3). It should be noted that several immune-related genes including CD19, IL7R, IL6, and CCR7 were upregulated in partial responders. A volcano plot with differentially expressed genes (linear fold change > 2 and *p* < 0.05) was used to compare SD+PD and PR (Fig. 4a). Decreased CDKN2A expression (*p* = 0.02) (Fig. 4b) and increased CALML5 (*p* = 0.01) (Fig. 4c) were associated with better response to therapy.

**Table 3:** NanoString PanCancer Pathways® differentially expressed genes

mRNA	Log2 fold change	Standard error (log2)	Linear fold change	*p* value	Gene sets
SPP1	− 2.96	0.804	0.129	0.0017	PI3K
CDKN2A	− 2.05	0.805	0.242	0.0211	Cell cycle, apoptosis, tumor suppressor gene
CNTFR	− 1.98	0.702	0.253	0.0111	JAK-STAT
DDIT4	− 1.65	0.557	0.319	0.0848	PI3K
WNT5A	− 1.49	0.702	0.355	0.0474	Hedgehog, Wnt
ITGA9	1.01	0.36	2.02	0.0116	PI3K
LAT	1.07	0.453	2.1	0.0297	Ras
IL7R	1.08	0.427	2.11	0.0214	JAK-STAT, PI3K
PPARGC1A	1.21	0.519	2.32	0.0312	Chromatin modification
TSLP	1.23	0.544	2.34	0.0379	JAK-STAT
ID4	1.31	0.353	2.47	0.00163	TGF-β
TNR	1.33	0.602	2.52	0.0408	PI3K
RASGRP2	1.42	0.614	2.68	0.0336	MAPK, Ras
HNF1A	1.49	0.591	2.8	0.0223	Driver gene
COL2A1	1.59	0.606	3.01	0.0178	PI3K
EFNA2	1.69	0.626	3.22	0.0154	PI3K, Ras
CCR7	1.7	0.68	3.25	0.0229	Transcriptional misregulation
IL2ORB	1.71	0.607	3.26	0.0116	JAK-STAT
WNT16	1.87	0.777	3.66	0.0277	Hedgehog, transcriptional misregulation, Wnt
CALML5	2.07	0.749	4.21	0.0127	Ras
IL6	2.51	0.707	5.71	0.00244	JAK-STAT, PI3K, transcriptional misregulation
CD19	2.62	0.764	6.13	0.00323	PI3K

Further analysis using NanoString BC360™ analysis was performed for 11 patients (due to limited sample). Biologically significant pathways and single gene expression results are shown in Additional file 1: Figure S1. Samples are grouped based on the initial stage (stage II, stage III, and de novo stage IV), lines of therapy > 2, PAM50 molecular subtypes (basal, HER2+, luminal A, and luminal B), and TNBC subtypes (BLIA, BLIS, LAR, and MES). BC360™ analysis revealed diverse tumor and tumor microenvironment (TME) pathway signatures with no observable pattern (Additional file 2: Figure S2A). There was no clear association of these variables and response to therapy, with the exception of HER2-enriched subtype which was associated with treatment resistance (*p* = 0.02) (Additional file 2: Figure S2B).

### Genomic mutation profiles

Nine patients had genomic mutation analysis through FoundationOne®. Additional file 3: Figure S3 shows the most frequent genomic alterations identified. Of the nine patients analyzed, two patients (3 and 16) carried mutations in the PI3K-Akt-mTOR pathway. Due to the limited sample size, the association of genomic alteration with clinical response or survival could not be assessed.

## Discussion

Activation of the *PI3K/AKT* pathway contributes to the resistance to anti-cancer agents including microtubule-targeting agents. Despite the high frequency of alterations of *PIK3CA/AKT/mTOR* pathway, the presence of these mutations does not translate to a significant response to single agent PI3K inhibitors in early clinical trials [43, 44]. This is likely attributed to multiple by-pass signaling pathways. We hypothesized that targeting both the microtubule cytoskeleton and the PI3K/AKT/mTOR pathway would lead to a synergistic anti-tumor effect. Our previous work showed synergistic inhibition of the PI3K/AKT/mTOR pathway, which resulted in an increased reduction of p-S6K1 and p-S6. The synergistic suppression of cell survival was found in a number of breast cancer cell lines in vitro and breast cancer mouse models in vivo [34].

The current study demonstrated that the combination of eribulin and everolimus is feasible and an effective treatment for patients with mTNBC. Everolimus 5 mg daily with eribulin 1.1 mg/m^2^ days 1 and 8 every 3 weeks was determined to be the RP2D doses [45]. Disease response rate was 36%. Tolerability of the combination improved after mandatory Dex mouthwash was added for dose level B1, which reconfirmed the SWISH data. Since 50% of DLTs in dose levels A1 and A2 were oral mucositis, the mandatory use of dexamethasone mouthwash may reduce DLTs in both dose levels.

Eribulin has been reported to have anti-tumor activity with a manageable tolerability profile with side effects consisting of neutropenia, fatigue, alopecia, nausea, and anemia. In addition, there was a low incidence of peripheral neuropathy [46–48]. The Eisai Metastatic Breast Cancer Study Assessing Physician’s Choice Verses E7389 (EMBRACE) clinical trial was a phase III trial of patients with heavily pretreated metastatic breast cancer. Participants received eribulin (E7389) monotherapy or treatment of physician’s choice (TPC). The patients in the trial received a median of four prior therapies. Improvement was seen in OS with HR 0.81, 95% CI [0.66–0.99], *p* = 0.041. Median OS was 13.1 months in patients receiving eribulin vs. 10.6 months in TPC [28]. This study led to the FDA approval of eribulin mesylate for the treatment of breast cancer in patients who had failed taxane- or anthracycline-based therapies.

The mTOR inhibitor everolimus has emerged as a potential combination therapy drug for the treatment of cancer unresponsive to conventional therapy [49]. When used alone, everolimus can induce increased levels of p-AKT via a negative feedback loop leading to resistance of cells to mTOR inhibitors [50, 51]. Dual blockade of mTOR and other PI3K pathway inhibitors results in synergistic decrease in cancer cell growth [33, 51, 52]. The PI3K pathway has been shown to play a critical role in TNBC. However, downstream of PI3K, mTOR inhibitor alone does not demonstrate clinical benefit. Studies combining chemotherapy with *PI3K/AKT/mTOR* pathway inhibitors have shown efficacy [38, 53, 54].

In a phase II trial of everolimus and carboplatin in metastatic TNBC, clinical benefit rate (CBR) was 36% and medical PFS was 3 months [38]. Other clinical trials have targeted different inhibitors in the PI3K pathway. Recently, the LOTUS trial studied the oral AKT inhibitor ipatasertib in TNBC. In this study, combination of ipatasertib and paclitaxel showed longer PFS compared to paclitaxel alone [53]. This improved PFS was observed particularly in patients with *PIK3CA/AKT1/PTEN*-altered tumors. The IPATunity130 trial is underway to confirm these findings [55].

In the NanoString analysis, we identified the following immune-related genes which were upregulated in patients who achieved partial responses: CD19, IL7R, IL6, and CCR7. Due to the limited sample size, the result is hypothesis-generating and requires further verification. This finding is consistent with the observation that upregulation of immune-related genes in TNBC correlates with better response to chemotherapy and improved survival [56, 57].

The current dose-defining study did not reveal the underlying mechanism predicting response to the combination of eribulin and everolimus. mRNA profiling and genomic analyses were performed, but with limited sample size. Although no clear conclusions were drawn, we observed an association between HER2-enriched subtype and poor response to therapy. Future studies targeting the PI3K-AKT-mTOR pathway will provide more insight into the molecular predictors of response.

## Conclusion

The combination of eribulin and everolimus is feasible and an effective treatment in metastatic TNBC. This phase I clinical trial defines the RP2D as eribulin 1.1 mg/m^2^ (days 1 and 8) and everolimus 5 mg daily for further study. A post hoc analysis showed that participants that used dexamethasone mouthwash stayed on treatment for one additional cycle.

## Supplementary information

**Additional file 1:** **Figure S1.** BC360™ analysis (*n*=11): A) Relevant gene signatures and biologically significant single genes are shown. Samples were grouped based on stage (stage II, stage III, and de novo stage IV), lines of therapy >2, molecular subtypes (PAM50: basal, HER2+, luminal A, and luminal B); and TNBC subtypes: BLIA, BLIS, LAR, and MES. Signatures scores are mapped to quantiles of TCGA with a 0.5 value approximating the median TCGA value.

**Additional file 2:** **Figure S2.** BC360™ analysis (n=11): A) Forest plot showing differentially expressed signatures comparing SD+PD and PR groups; B) HER2-enriched signature is up-regulated in SD+PD compared with PR group (*P*=0.02).

**Additional file 3:** **Figure S3.** Tile plot showing genomic mutations in mTNBC patients (*n*=9).

**Additional file 4:** **Table S1.** Normalized mRNA expression data for NanoString PanCancer Pathways® cohort (*n*=20).

**Additional file 5:** **Table S2.** Normalized mRNA expression data for NanoString BC360™ cohort (n=11).

**Additional file 6:** **Table S3.** Genomic alteration data for TNBC patients with genomic reports (n=9).

## Data Availability

Normalized mRNA expression data for NanoString PanCancer cohort is available in Additional file 4: Table S1, normalized mRNA expression data for the NanoString BC360 cohort is available in Additional file 5: Table S2, and available genomic alteration data is available in Additional file 6: Table S3.

## Abbreviations

TNBC: Triple-negative breast cancer
HER-2: Human epidermal growth factor receptor amplification
mTNBC: Metastatic TNBC
OS: Overall survival
PARP: Poly ADP ribose polymerase
PD-1: Programmed death 1
PD-L1: Programmed death ligand 1
PIK3CA: Phosphatidylinositol-4, 5-bisphosphate 3-kinase, catalytic subunit, alpha
PTEN: Phosphatase and tensin homolog
EMT: Epithelial-mesenchymal transition
MET: Mesenchymal-epithelial transition
mTOR: Mammalian target of rapamycin
RP2D: Recommended phase II dose
RR: Response rate
PFS: Progression-free survival
ANC: Absolute neutrophil count
UNL: Upper limit of normal
TEQR: Toxicity equivalence range
DLTs: Dose-limiting toxicities
MTD: Maximum tolerated dose
