# Supplementary figures and images for: Phase I clinical trial of the combination of eribulin and everolimus in patients with metastatic triple-negative breast cancer
Source: Breast Cancer Res. 2019 Nov 8;21:119. doi: 10.1186/s13058-019-1202-4 (PMC6839083; doi:10.1186/s13058-019-1202-4)

A

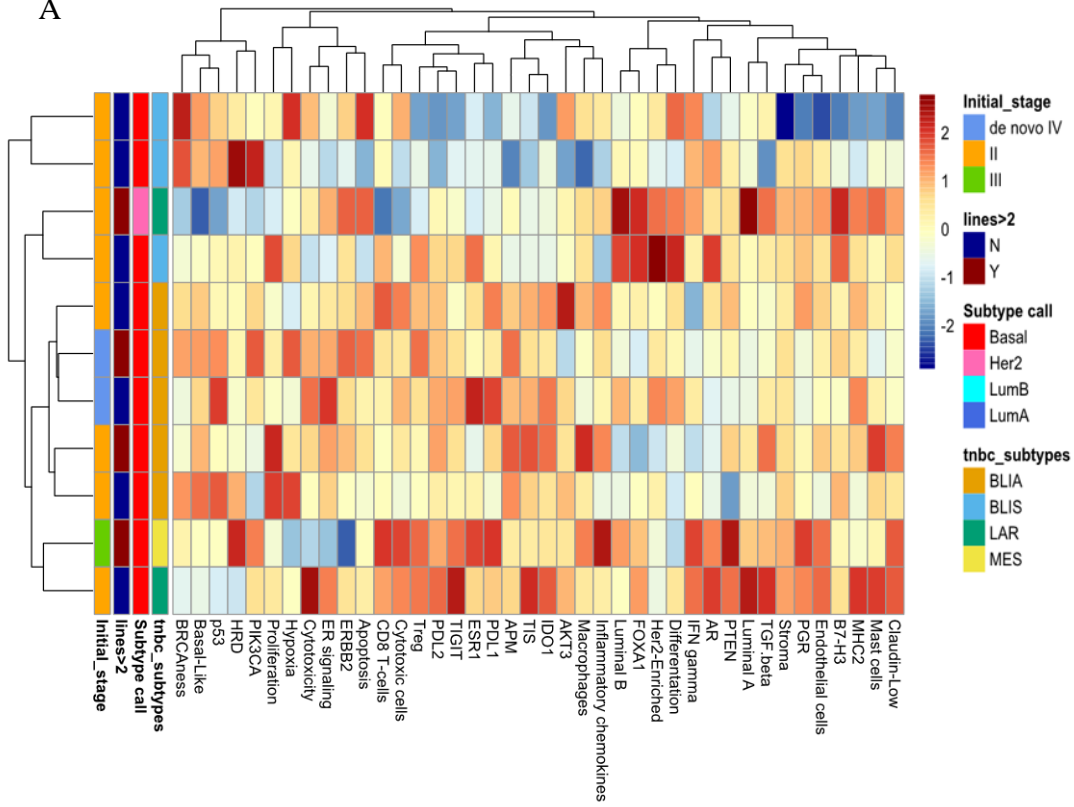

Supplement: Supplementary file 1 — Additional file 1: Figure S1. BC360™ analysis (n=11): A) Relevant gene signatures and biologically significant single genes are shown. Samples were grouped based on stage (stage II, stage III, and de novo stage IV), lines of therapy >2, molecular subtypes (PAM50: basal, HER2+, luminal A, and luminal B); and TNBC subtypes: BLIA, BLIS, LAR, and MES. Signatures scores are mapped to quantiles of TCGA with a 0.5 value approximating the median TCGA value. [file 13058_2019_1202_MOESM1_ESM.pdf]

**A**

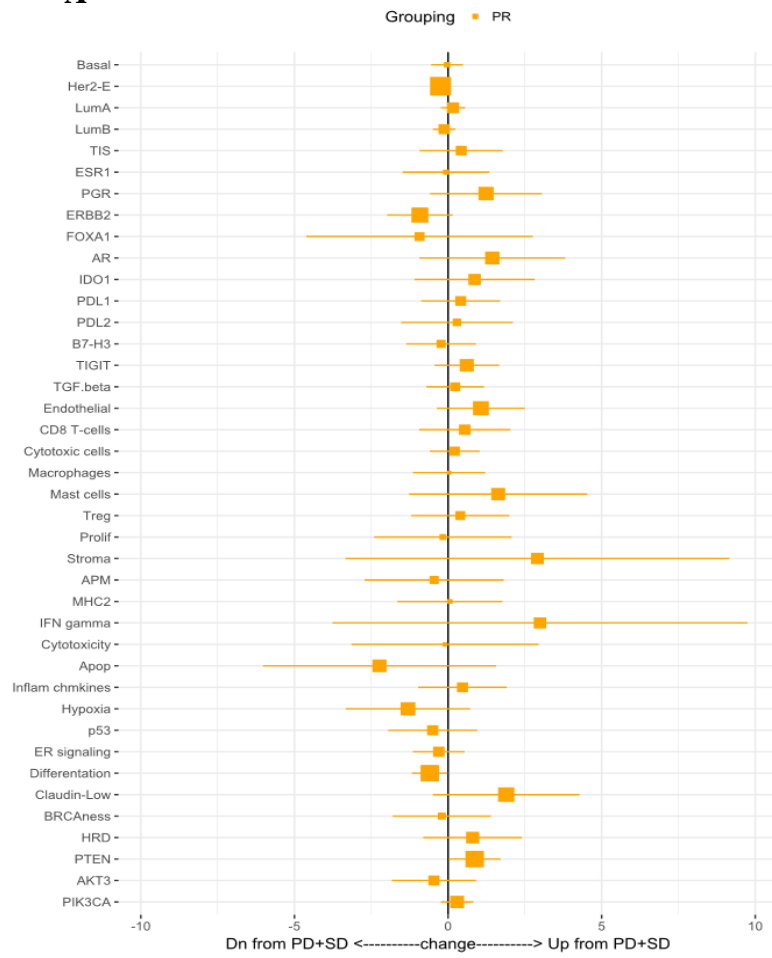

**B**

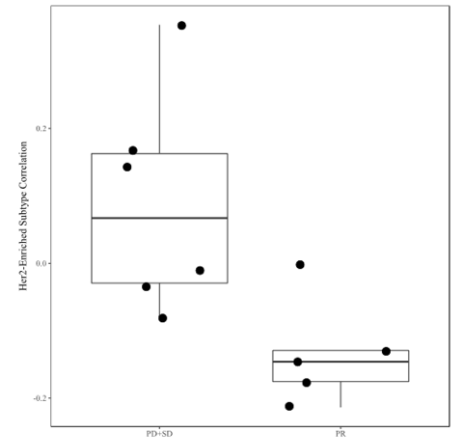

Supplement: Supplementary file 2 — Additional file 2: Figure S2. BC360™ analysis (n=11): A) Forest plot showing differentially expressed signatures comparing SD+PD and PR groups; B) HER2-enriched signature is up-regulated in SD+PD compared with PR group (P=0.02). [file 13058_2019_1202_MOESM2_ESM.pdf]

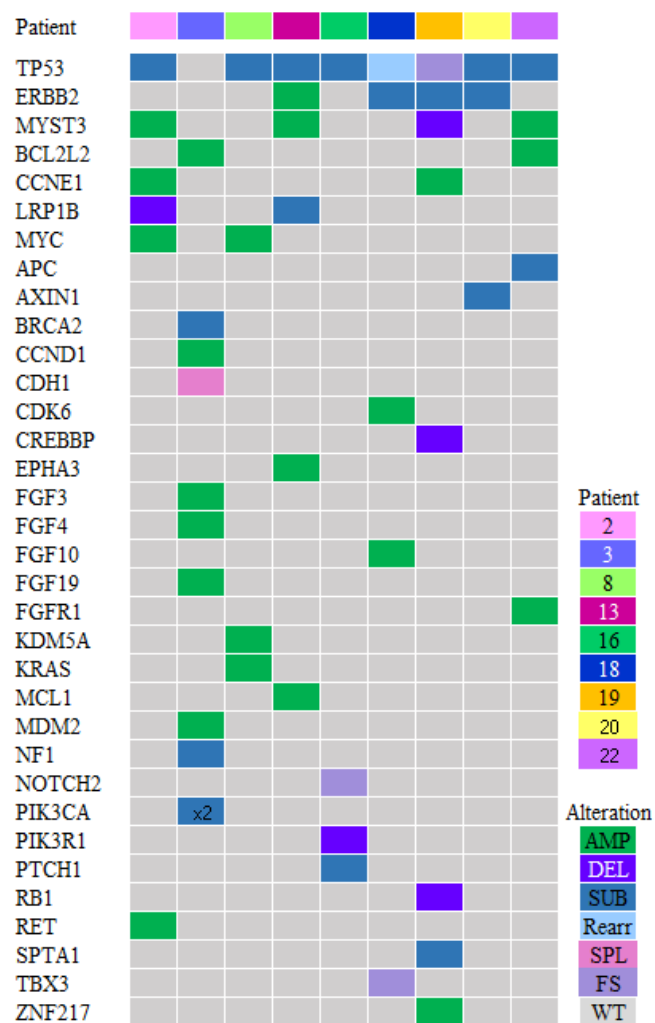

Supplement: Supplementary file 3 — Additional file 3: Figure S3. Tile plot showing genomic mutations in mTNBC patients (n=9). [file 13058_2019_1202_MOESM3_ESM.pdf]
